# Mitochondria-targeted antioxidant MitoQ ameliorates experimental mouse colitis by suppressing NLRP3 inflammasome-mediated inflammatory cytokines

**DOI:** 10.1186/1741-7015-11-178

**Published:** 2013-08-06

**Authors:** Amarjargal Dashdorj, Jyothi KR, Sangbin Lim, Ara Jo, Minh Nam Nguyen, Joohun Ha, Kyung-Sik Yoon, Hyo Jong Kim, Jae-Hoon Park, Michael P Murphy, Sung Soo Kim

**Affiliations:** 1 Department of Biochemistry and Molecular Biology, School of Medicine, Kyung Hee University, Seoul 130-701, Republic of Korea; 2 Division of Gastroenterology, Department of Internal Medicine, School of Medicine, Kyung Hee University, Seoul 130-701, Republic of Korea; 3 Department of Pathology, School of Medicine, Kyung Hee University, Seoul 130-701, Republic of Korea; 4 MRC Mitochondrial Biology Unit, Hills Road, Cambridge CB2 0XY, UK

**Keywords:** Dextran sulfate sodium, Interleukin-1 beta, Interleukin-18, MitoQ;NLRP-3 inflammasome, Reactive oxygen species

## Abstract

**Background:**

MitoQ is a mitochondria-targeted derivative of the antioxidant ubiquinone, with antioxidant and anti-apoptotic functions. Reactive oxygen species are involved in many inflammatory diseases including inflammatory bowel disease. In this study, we assessed the therapeutic effects of MitoQ in a mouse model of experimental colitis and investigated the possible mechanisms underlying its effects on intestinal inflammation.

**Methods:**

Reactive oxygen species levels and mitochondrial function were measured in blood mononuclear cells of patients with inflammatory bowel disease. The effects of MitoQ were evaluated in a dextran sulfate sodium-induced colitis mouse model. Clinical and pathological markers of disease severity and oxidative injury, and levels of inflammatory cytokines in mouse colonic tissue were measured. The effect of MitoQ on inflammatory cytokines released in the human macrophage-like cell line THP-1 was also analyzed.

**Results:**

Cellular and mitochondrial reactive oxygen species levels in mononuclear cells were significantly higher in patients with inflammatory bowel disease (*P* <0.003, cellular reactive oxygen species; *P* <0.001, mitochondrial reactive oxygen species). MitoQ significantly ameliorated colitis in the dextran sulfate sodium-induced mouse model *in vivo*, reduced the increased oxidative stress response (malondialdehyde and 3-nitrotyrosine formation), and suppressed mitochondrial and histopathological injury by decreasing levels of inflammatory cytokines IL-1 beta and IL-18 (*P <*0*.*001 and *P* <0.01 respectively). By decreasing mitochondrial reactive oxygen species, MitoQ also suppressed activation of the NLRP3 inflammasome that was responsible for maturation of IL-1 beta and IL-18. *In vitro* studies demonstrated that MitoQ decreases IL-1 beta and IL-18 production in human THP-1 cells.

**Conclusion:**

Taken together, our results suggest that MitoQ may have potential as a novel therapeutic agent for the treatment of acute phases of inflammatory bowel disease.

## Background

Inflammatory bowel disease (IBD) is a chronic relapsing inflammatory disorder of the intestine that comprises two major clinical forms, namely, ulcerative colitis (UC) and Crohn’s disease (CD). Clinical features consist of diarrhea, abdominal pain, rectal bleeding and weight loss. The complications observed in colonic manifestations are bowel strictures, obstruction, abscess formation and perforation. In addition, IBD leads to extra-colonic inflammatory manifestations in many organs including the joints, eyes, skin and liver [1]. Conventional medications such as corticosteroids and immune-modulators are the first line of therapy for IBD. However, remission from corticosteroids is only maintained for a short period, and treatment impairs general immunity. A recently developed monoclonal antibody targeting TNF-α shows efficacy in inducing and maintaining remission, but has serious side effects, including an increased risk of infection. Moreover, some patients do not show adequate responses [2,3]. Systematic review of population-based studies between 1950 and 2010 shows that prevalence and incidence of IBD are dramatically increasing throughout the world [4]. Therefore, there is a need to develop safer and more effective therapies for IBD.

The exact etiology of IBD is still not fully understood, but dysfunctional immunoregulation of the gut plays a crucial role in the pathogenesis of IBD [5]. IL-1 beta and IL-18 are the major pro-inflammatory cytokines that promote activation of both the innate and adaptive immune responses [6,7]. Hypoxia-induced transcription factor forkhead box p3 (Foxp3) enhances regulatory T cells, which are essential for immune tolerance and play a crucial role in the limitation of excessive helper T cells induction and inflammatory response [8]. But, inflammatory cytokines including IL-1 beta inhibit Foxp3 function, induce differentiation of helper T cells, and can cause T cell-mediated inflammation [9,10]. High levels of IL-1 beta and IL-18 expression in patients with IBD [11,12] and their correlation with disease activity [13] have been well described, and indicate that these cytokines play an important role in promoting localized inflammation in IBD. IL-1 beta and IL-18 are expressed as inactive precursors and activated after cleavage by the NACHT, LRR and PYD domains-containing protein 3 (NLRP3) inflammasome, whose mutations have been associated with CD [14]. The NLRP3 inflammasome is a multi-protein, caspase-1 activating complex, and its dysregulation is strongly associated with many inflammatory diseases [15]. Several reports have shown that the NLRP3 inflammasome plays a pathological role in experimental colitis [16,17], and that activation of the NLRP3 inflammasome is mediated by mitochondrial reactive oxygen species (mtROS) [18-20]. Moreover, many studies have shown that ROS mediate intestinal tissue injury, and that administration of antioxidants or overexpression of antioxidant enzymes leads to amelioration of experimental colitis. Furthermore, a strong association between oxidative stress and IBD has been observed in many human studies [21]. A possible role of mitochondrial dysfunction in the pathogenesis of IBD was reported in clinical cases [22,23], and these data also suggested that mtROS play a role in the pathogenesis of IBD.

MitoQ is an orally available mitochondria-targeted derivative of the antioxidant ubiquinone. MitoQ comprises a lipophilic triphenylphosphonium (TPP) cation that drives rapid permeation of phospholipid bilayers and leads to an accumulation within mitochondria. In the mitochondrial matrix, MitoQ is continually reduced by the respiratory chain to its active form and protects mitochondria from oxidative damage. Due to the characteristic of selective accumulation and continual recycling within mitochondria, MitoQ has been demonstrated *in vitro* and *in vivo* to be protective against many oxidative damage-related pathologies, including ischemia-reperfusion injury [24], cardiovascular diseases [25,26], ethanol-dependent hepatosteatosis [27] and sepsis [28]. MitoQ has successfully been tested in phase I and phase II clinical trials and shown to be effective against liver damage in patients with hepatitis C infection [29]. Importantly, these clinical trials showed that MitoQ has no severe adverse effects.

In this study, we hypothesized that MitoQ might decrease the excessive activation of the NLRP3 inflammasome, and thus attenuate acute phases of IBD. Therefore, we tested if MitoQ can function as a therapeutic agent to treat acute colonic injury in a mouse model of dextran sulfate sodium (DSS)-induced colitis.

## Methods

### Peripheral blood mononuclear cells

Blood samples were obtained from seven patients with active CD (four men, three women), seven with active UC (five men, two women) and 14 healthy volunteers (five women, nine men) as normal controls. Blood samples were carefully layered over 3 to 5 ml of polymorphonuclear leukocyte isolation medium (Cedarlane Laboratories, Hornby, ON, Canada). Samples were centrifuged at 450 g for 30 minutes at 18°C. At the end of centrifugation, the top band that consisted of mononuclear cells (MNC) was harvested with a Pasteur pipette, repeatedly washed with Hank’s balanced salt solution and then subjected to ROS levels measurement and preparation of mitochondrial proteins. This study was approved by the Institutional Review Board of the Kyung Hee University of Korea, College of Medicine. All participants signed a written informed consent form before any protocol-specific procedure was carried out.

### Reactive oxygen species analysis

ROS levels were measured with the fluorescent probes 2ʹ, 7ʹ–dichlorofluoresceindiacetate (DCF-DA) and MitoSOX. Cells were loaded with 10 μM of DCF-DA or MitoSOX at 37°C for 30 minutes and washed with 1 ml PBS. Fluorescence was determined with excitation at 488 nm and emission at 525 nm by flow cytometer (FACSCalibur; Becton-Dickinson, Franklin Lakes, NJ, USA).

### Preparation of mitochondrial fraction and mitochondrial proteins

Preparation of mitochondrial fraction and proteins were performed as previously described [30]. The isolated mitochondria were subjected to mitochondrial electron transport chain complex expression measurements as previously described [31].

### Animals

Female Balb/c (wild type; WT) mice (6 to 7 weeks of age; weighing 18 to 20 g) were purchased from Central Lab. Animal, Inc. (Seoul, Korea). Four to five animals were housed per cage and fed standard mice chow pellets, had access to tap water supplied in bottles, and were acclimatized 7 days before they went into experiments. The experimental protocol was approved by the Institutional Animal Care and Use Committee of Kyung Hee University (Seoul, Korea).

### Induction of colitis and treatment

DSS(molecular weight, 36,000-50,000) was purchased from MP Biomedicals (Illkirch, France). Mice were divided into four groups: control group (WT), DSS-induced colitis group (WT+DSS), DSS with decyltriphenylphosphonium bromide (dTPP)-treated group (DSS+dTPP), and DSS with MitoQ-treated (DSS+MitoQ) group. Considering the fast recovery of DSS-induced colitis in mice after DSS withdrawal [32], we gave 4% DSS in their drinking water from day 0 to day 7, followed by 1% DSS for maintaining pathology as described previously [33]. None of the mice in this study died before termination of the experiment at day 21. Control mice were given tap water. MitoQ and dTPP were administered orally at a final concentration of 500 μM from day 7 for 14 days until the end of experiments [34]. All compounds were dissolved in water and given fresh every third day. Clinical scores of colitis, such as weight change and colorectal bleeding, were observed. Mice were then sacrificed and the colons were removed, cleaned, and length was measured.

### Isolation of peritoneal macrophages

Mice were anesthetized with isoflurane, sacrificed by cervical dislocation, and injected with 10 ml of PBS. After 30 seconds of abdominal massaging, peritoneal lavage was performed. Collected peritoneal lavage was centrifuged and plated in 60 well plates and incubated for 2 hours. Adherent cells were analyzed in subsequent experiments.

### Histological analysis

Distal colonic sections of 1.5 cm were fixed in 10% neutral-buffered formalin, processed for paraffin embedding, sectioned at 5 μm and stained with hematoxylin and eosin according to standard protocols. Histological scoring was performed in a blinded fashion by a pathologist using a combined score of inflammatory cell infiltration (score 0 to 3) and tissue damage (score 0 to 3). Focally increased numbers of inflammatory cells in the lamina propria were scored as 1, confluence of inflammatory cells extending into the submucosa as 2, and transmural extension of the infiltrate as 3. For tissue damage, discrete lymphoepithelial lesions were scored as 1, mucosal erosions as 2, and extensive mucosal damage or extension through deeper structures of the bowel as 3. The two equally measured sub-scores were added and the combined histological colitis severity ranged from 0 to 6.

### Transmission electron microscopy

Colon tissues were fixed in fixative solution (2% glutaraldehyde and 1% formaldehyde in 0.1 M sodium cacodylate buffer, pH 7.4) for 2 hours and washed with sodium cacodylate buffer. After fixing with 1% osmium tetraoxide, tissues were washed and dehydrated by replacement of ascending series of alcohol. Tissues were embedded in epon and propylene oxide (1:1) and sectioned using an ultramicrotome (Reichert Ultracut S, Leica Microsystems, Wetzlar, Germany). Ultrastructural changes were observed using a transmission electron microscope (Zeiss EM 902A, Oberkohen, Germany) under 80 kV.

### Immunohistochemical analysis

Colons were fixed in 10% buffered formalin, dehydrated, embedded in paraffin and sectioned at 5 μm slices. Sections were stained with anti-malondialdehyde antibody (Genox Corp., Baltimore, MD, USA) or anti-nitrotyrosine antibody (Cayman Chemical, Ann Arbor, MI, USA) overnight, followed by incubation with biotin-labeled anti-rabbit antibody. Both sections were counterstained by hematoxylin (Gene Tex, Irvine, California, USA) and mounted with (Vector laboratories, Burlingame, CA, USA). The immunostained sections were visualized with an EnVision Detection Kit (Dako, CA, USA).

### Cytokine measurement

Colon homogenates were centrifuged at 15,000 rpm for 15 minutes. The amounts of IL-1 beta and IL-18 were quantified by ELISA (MBL and R&D Systems, Minneapolis, MN, USA) according to the manufacturer’s protocol.

### Reverse transcription-PCR

For the analysis of IL-1 beta and IL-18 mRNA, total RNA was extracted from colon tissue using Trizol reagent (Invitrogen, Carlsbad, CA, USA) and 1 μg was amplified using the following specific primers: IL-1 beta forward, 5’-ACAACTGCACTACAGGCTCC-3’, and reverse, 5’-CTCTGCTTGTGAGGTGCTGA-3’; IL-18 forward, 5’-GGCTGCCATGTCAGAAGACT-3’, and reverse, 5’-GTCTGGTCTGGGGTTCACTG-3’; GAPDH forward, 5’-CAACTTTGGCATTGTGGAAGGG-3’, and reverse, 5’-ACACATTGGGGGTAGGAACA-3’.

The amplified products were visualized on a 1% agarose gel, and the amplified GAPDH fragment was used as an internal control for RT-PCR.

### Cell culture

Human THP-1 cells were grown in Roswell Park Memorial Institute medium, supplemented with 10% fetal bovine serum, 100 units/ml of penicillin and 100 μg/ml of streptomycin. Cells were differentiated with 100 nM phorbol 12-myristate 13-acetate (Sigma Chemical Co, St Louis, MO, USA) for 24 hours. Cells were then treated with H_2_O_2_ (5 mM) for 6 hours with or without MitoQ (50 to 150 nM). Before collecting supernatants, cells were subsequently stimulated by ATP for 30 minutes. Supernatants and cell lysates were analyzed in subsequent experiments.

### Immunoprecipitation and western blotting analysis

Immunoprecipitation (IP) was performed as described previously [35]. The IP samples and colon homogenates were separated using 8% to 13.5% SDS-polyacrylamide gels. The following primary antibodies were used: each subunit of mitochondrial complexes (MitoScience, Eugene, OR, USA), NLRP3 (mouse monoclonal; Adipogen, Inc., Incheon, Korea), apoptosis-associated speck-like protein containing a CARD (ASC; rabbit monoclonal, Adipogen, Inc.), caspase-1 (rabbit polyclonal; Santa Cruz Biotechnology, Santa Cruz, CA, USA), IL-18 (mouse monoclonal; R&D Systems), IL-1 beta (rabbit polyclonal; BioVision, Inc., Milpitas, CA, USA),thioredoxin (TRX;rabbit monoclonal; AbFrontier, Seoul, Korea), TRX interacting protein (TXNIP; goat monoclonal; MBL International, Woburn, MA, USA) and actin (goat polyclonal; Abcam, Cambridge, MA, USA). Blots were washed with Tris-buffered saline with Polyethylene glycol sorbitan monolaurate 20 and developed with enhanced chemiluminescence reagents (Santa Cruz Biotechnology).

### Statistical analysis

Results were expressed as mean ± standard error (SE). Error bars represent the mean ±SE of at least three independent experiments. The difference between two mean values was analyzed using a Student’s t-test. The difference was considered statistically significant when *P* <0.05.

## Results

### Reactive oxygen species levels increase in the mononuclear cells of patients with inflammatory bowel disease

**Figure 1:**
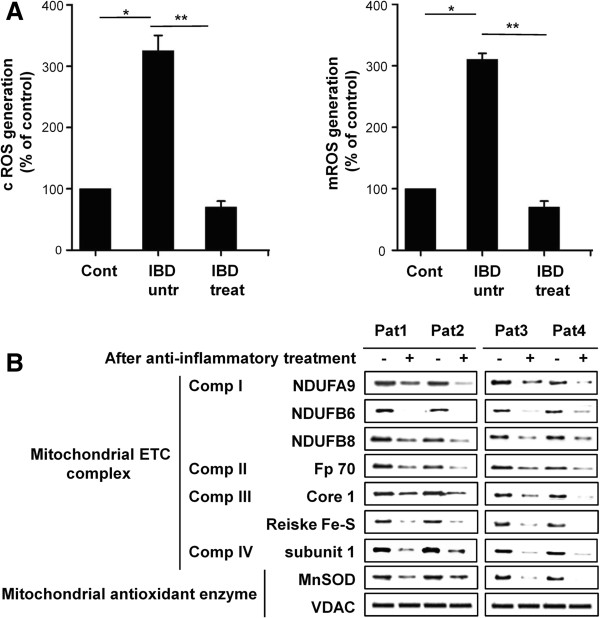
**Mitochondrial reactive oxygen species generation in patients with inflammatory bowel disease. (A)** Intracellular H_2_O_2_ and mitochondrial peroxynitrate levels in mononuclear cells of patients with IBD before (IBD untr) and after treatment (IBD treat), compared with healthy volunteers (cont). Results are expressed as means ±SE. *P <0.003, **P <0.001. **(B)** Western blotting analysis of the expression patterns of various mtETC complex subunits from mitochondria isolated from peripheral blood MNC of patients. pat – patient. IBD, inflammatory bowel disease; mtETC, mitochondrial electron transport chain; MNC, mononuclear cells.

To determine whether there was an increase in ROS during IBD, we measured ROS levels via flow cytometry using the ROS probes DCF-DA and MitoSOX in the MNC of patients with IBD. Blood samples were obtained from patients before and after treatment. Patients received standard drugs in combination, as illustrated in Additional file 1: Table S1. Interestingly, ROS levels, as measured by both probes, were increased in the MNC of patients with active IBD. However, ROS levels were significantly decreased in patients in clinical remission (Figure 1A). To assess whether the changes in ROS levels were associated with changes in mitochondrial function, we checked the expression levels of mitochondrial electron transport chain (mtETC) complex subunits. As shown in Figure 1B, expression levels of mtETC complexes were increased during IBD, but decreased after treatment and clinical remission was achieved. We also checked induction of manganese superoxide dismutase, which responds to elevated oxidative stress in mitochondria. The expression level of MnSOD was increased during IBD and decreased after treatment. Voltage dependent anion channel was used to verify that mitochondrial proteins were equally loaded for western blotting analysis. From these results, we concluded that changes in mitochondrial function and mtROS levels correlate with IBD.

### MitoQ attenuates dextran sulfate sodium-induced colitis

**Figure 2:**
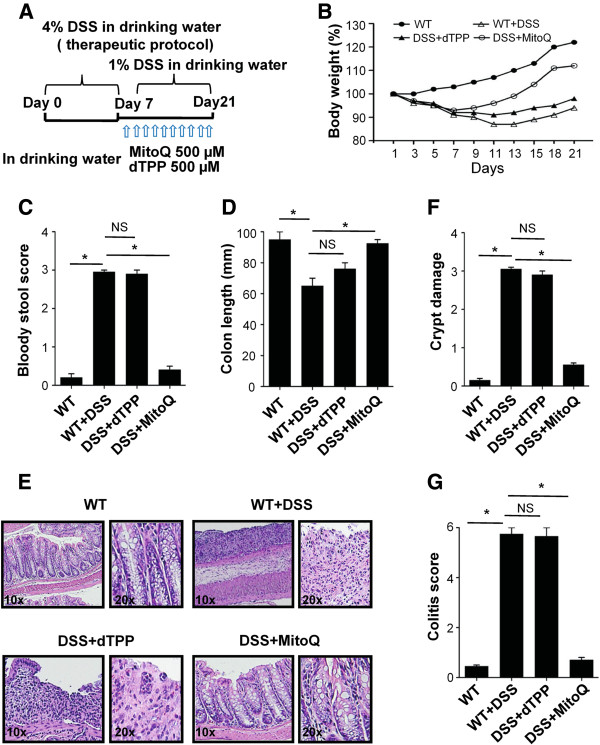
**Therapeutic potential of MitoQ for dextran sulfate sodium-induced colitis. (A)** Experimental design for DSS-induced colitis in mice. 4% DSS was administered to WT mice from day 0 to day 7 followed by 1% of DSS for the duration of the experiment. Two groups of mice additionally received dTPP and MitoQ from day 7. **(B)** Body weight of mice was measured every 3 days and presented as a percentage of their initial weight, n = 5 mice per group. **(C)** Bloody stool score on the 10^th^ day. **(D)** Lengths of the freshly removed colons were measured from rectum to ileocecal junction. **(E)** Representative distal colon sections stained with hematoxylin and eosin. The magnification is indicated. **(F)** Crypt damage. **(G)** Colitis score. For all samples, results are expressed as mean ±SE. n = 3, *P <0.001. DSS+dTPP, DSS with dTPP-treated mice; DSS+MitoQ, DSS with MitoQ-treated mice; NS, not significant; WT, control mice; WT+DSS, DSS-treated mice.

Since elevated ROS levels and changes in mitochondrial function seemed to correlate with the pathogenesis of IBD, we investigated the therapeutic effect of MitoQ on DSS-induced mouse colitis. To induce severe colitis, we treated mice with 4% DSS for 7 days and then 1% DSS for another 14 days in their drinking water. MitoQ or dTPP was administered from day 7 until the end of the experiment (Figure 2A). dTPP, which contains the same lipophilic cation as MitoQ but lacks antioxidant activity, was used as a negative control. Body weight loss was significantly increased in mice with DSS-induced colitis, and treatment with dTPP did not reverse this weight loss. However, mice with DSS-induced colitis treated with MitoQ gained weight similar to control mice (Figure 2B). The colon length shortening and bloody stool score were also significantly increased in mice treated with DSS or DSS+dTPP. Once again, MitoQ administration inhibited the DSS-induced bloody stool and decreased the colon length shortening (Figure 2C,D). Distal colonic sections from DSS and DSS+dTPP-treated mice revealed multifocal inflammatory cell infiltration and edema with crypt and epithelial cell destruction and ulceration. By contrast, no mucosal inflammation was observed in colonic sections of DSS+MitoQ-treated mice (Figure 2E,F). Colitis score was also significantly lower in MitoQ-treated colitis mice than in the DSS and DSS+dTPP-treated mice (Figure 2G). These data reveal that MitoQ inhibits clinical and histological changes in the colon associated with DSS-induced colitis.

### MitoQ attenuates mitochondrial injury and oxidative damage

**Figure 3:**
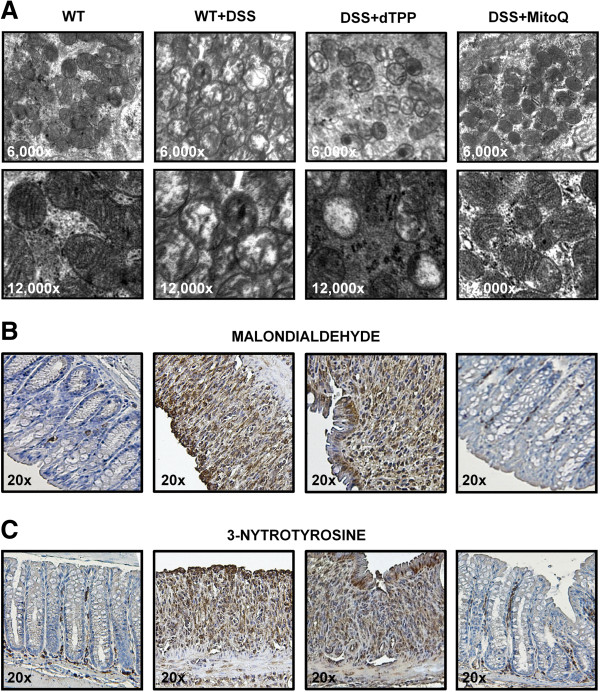
**MitoQ attenuates mitochondrial alteration and oxidative damage during DSS-induced colitis. (A)** Representative transmission electron micrographs of colon. The magnification is indicated. Similar histological profiles were seen in three separate colons per group. **(B)** Sections of colonic tissues were subjected to immunohistochemical analysis with an antibody against malondialdehyde. The magnification is indicated. **(C)** Sections of colonic tissues were subjected to immunohistochemical analysis with an antibody against 3-nitrotyrosine staining. The magnification is indicated.

To demonstrate the effect of MitoQ on mitochondria during colitis, we first studied mitochondrial structural changes. Electron microscopy of the colon of control mice revealed good preservation of normal mitochondrial structure (Figure 3A, left). In colon tissue in DSS and DSS+dTPP-treated mice,the majority of mitochondria had alterations in size and matrix. In some mitochondria, the matrix totally disappeared and only the outer membrane remained. In others, the cristae were disorganized because of edema in the matrix (Figure 3A, middle). MitoQ treatment reduced the morphological and mitochondrial injury during colitis (Figure 3A, right). There was a significant increase of malondialdehyde formation (a marker of lipid peroxidation, brown staining) in the colon during colitis (Figure 3B, middle) and MitoQ dramatically reduced the malondialdehyde formation (Figure 3B, right). Colitis was also associated with increased mitochondrial nitrotyrosine formation in the colon, an index of peroxynitrate-mediated protein nitration (Figure 3C, middle). However, MitoQ was protective against nitrate damage of the colon during colitis, as shown in Figure 3C (right). These data reveal that MitoQ protects mitochondria and reduces oxidative damage in the colon of mice with DSS-induced colitis.

### MitoQ inhibits caspase-1 activation through suppression of TXNIP binding to NLRP3 during colitis

**Figure 4:**
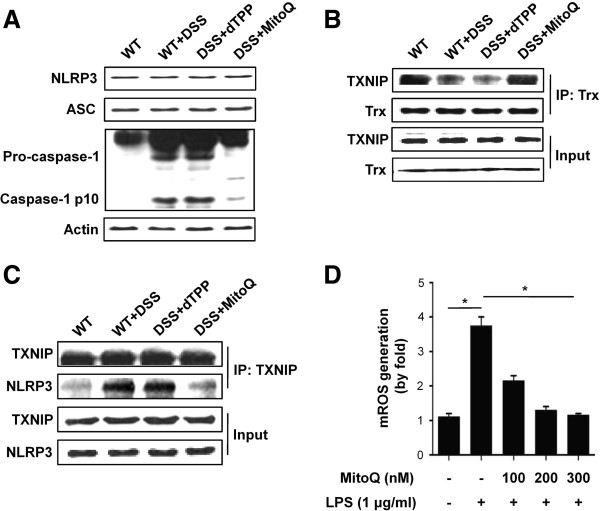
**MitoQ inhibits caspase-1 activation through suppression of TXNIP binding to NLRP3 during colitis. (A)** Western blotting analysis reveals expression of the NLRP3 inflammasome components in mouse colon homogenates. **(B)** Dissociation of the TXNIP-TRX complex upon MitoQ treatment, as revealed by co-immunoprecipitation. **(C)** Co-immunoprecipitation and western blotting analysis identify the interaction of TXNIP with NLRP3. IP, immunoprecipitation; input of cell extract without immunoprecipitation ensures equal loading. **(D)** Effect of MitoQ on mtROS production in peritoneal macrophages. Results are expressed as mean ±SE. n= 5. *P <0.01.

To clarify the mechanism of MitoQ in attenuation of colitis, we investigated the function of the NLRP3 inflammasome. The NLRP3 inflammasome comprises adaptor proteins ACS and caspase-1. It isknown that TXNIP binds to the leucine-rich repeat domain of NLRP3 and activates the inflammasome during oxidative stress [20]. TXNIP binds to TRX and negatively regulates its redox function in resting cells [36]. Oxidized TRX during oxidative stress is dissociated from the TXNIP-TRX complex, and leads to the interaction of TXNIP with NLRP3. Therefore, we hypothesized that binding of TXNIP to NLRP3 activates the inflammasome, which causes autocleavage of caspase-1 and the release of mature cytokines IL-1 beta and IL-18 during colitis. Western blotting analysis revealed that expression of the inflammasome complex proteins such as NLRP3 and ASC are not changed during colitis, but procaspase-1 is increased in its expression and cleaved into caspase-1 in DSS- and DSS+dTPP-treated mice. However, procaspase-1 was not cleaved in control and MitoQ-treated mice (Figure 4A). Next, we performed co-IP to check the interaction between TXNIP and NLRP3, and revealed that TXNIP is dissociated from TRX in DSS- and DSS+dTPP-treated mice (Figure 4B). During colitis, the dissociated TXNIP was bound to NLRP3, and this interaction was blocked by MitoQ treatment (Figure 4C). To gain more insight into the mechanism of oxidative stress-induced colitis, we isolated mouse peritoneal macrophages and measured mitochondrial ROS levels. Macrophages of DSS+MitoQ-treated mice released lower levels of ROS compared with macrophages of DSS- and DSS+dTPP-treated mice (Figure 4D). Therefore, we concluded that activation of NLRP3 inflammasome during colitis is dependent on the interaction of TXNIP and NLRP3, and that this activation is mediated by mtROS.

### MitoQ suppresses increased levels of pro-inflammatory cytokines IL-1 beta and IL-18 during colitis

**Figure 5:**
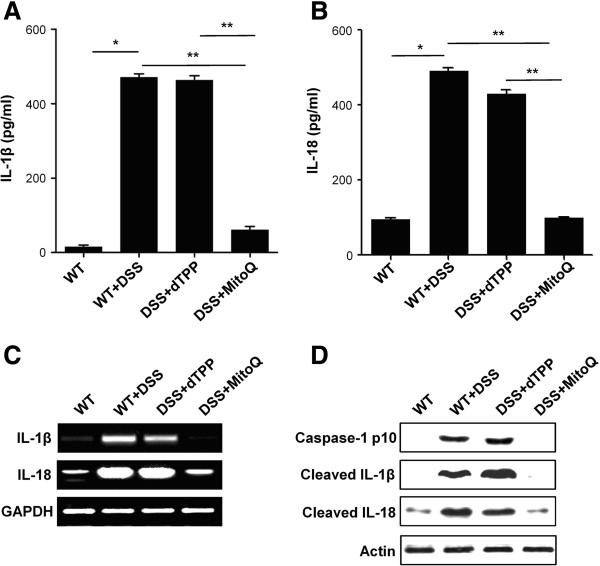
**MitoQ suppresses caspase-1-mediated IL-1 beta and IL-18 release during colitis.** ELISA assay for **(A)** IL-1 beta or **(B)** IL-18 were performed in colon homogenates. Results are expressed as mean ±SE. n=5. *P <0*.*001,**P<0.01. **(C)** mRNA expression levels of IL-1 beta and IL-18 in colon tissue were examined by RT-PCR. **(D)** Cleavages of caspase-1, IL-1 beta and IL-18 were analyzed by western blotting analysis in colon homogenates.

We next asked whether an activated inflammasome leads to enhanced release of IL-1 beta and IL-18 during colitis. Release of the active inflammatory cytokines IL-1 beta and IL-18 is mediated by a two-step process: first, recognition of pro-inflammatory signals by pattern recognition receptors on host cells and activation of pro-IL-1 beta and pro-IL-18 promoters; second, activation of the inflammasome by danger signals, resulting in activation of caspase-1 and cleavage of pro-IL-1 beta and pro-IL-18 [37]. Therefore, we evaluated how MitoQ affects the release of these cytokines in colon homogenates. Levels of IL-1 beta and IL-18 were significantly higher in DSS- and DSS+dTPP-treated mice than in control and DSS+MitoQ-treated mice, suggesting that MitoQ suppresses the release of these cytokines (Figure 5A,B). Furthermore, the mRNA levels of IL-1 beta and IL-18 were higher in DSS- and DSS+dTPP-treated mice, but suppressed with MitoQ treatment (Figure 5C). Lastly, western blotting analysis demonstrated the increased level of cleaved forms of caspase-1, IL-1 beta and IL-18 in the colon of DSS- and DSS+dTPP-treated mice, but the decreased cleavage in control and DSS+MitoQ-treated mice (Figure 5D). These data clearly demonstrate that MitoQ not only suppresses release of the active forms of IL-1 beta and IL-18, but also their transcriptional up-regulation.

### MitoQ decreases the levels of IL-1 beta and IL-18 in a human macrophage cell line

**Figure 6:**
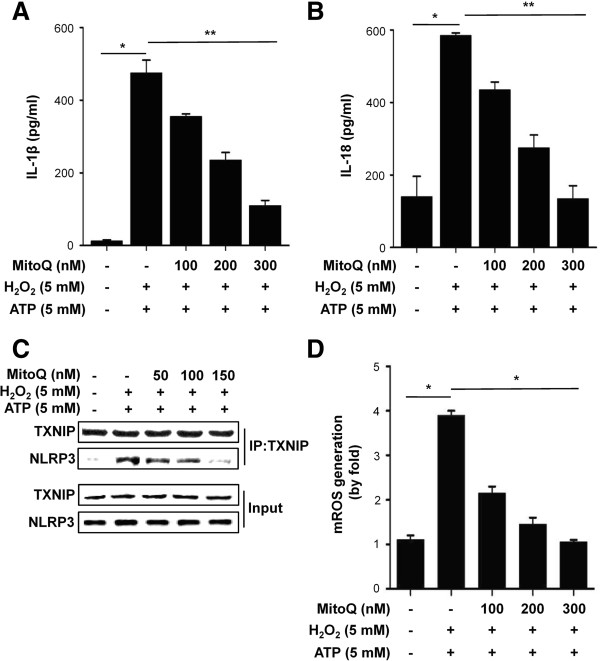
**Effect of MitoQ on human THP-1 cells. (A)** IL-1 beta and **(B)** IL-18 release in response to H_2_O_2_ or ATP were analyzed by ELISA. Results are expressed as mean ±SE. n=5,*P<0.001. **(C)** The interaction between TXNIP and NLRP3 was examined by co-IP and western blotting analysis. **(D)** mtROS production in THP-1 cells. Cells were differentiated for 24 hours with 100 nM phorbol 12-myristate 13-acetate. Results are expressed as mean ±SE. *P<0.001.

Finally, we investigated the *in vitro* effect of MitoQ on IL-1 beta and IL-18 production in a human macrophage-like cell line, THP-1. ELISA analysis revealed that MitoQ dose-dependently reduces the release of these cytokines whereas it is induced by H_2_O_2_ and ATP (Figure 6A,B). Furthermore, co-IP studies revealed that TXNIP is dissociated from TRX and binds to NLRP3, and this interaction is blocked by MitoQ treatment (Figure 6C). Lastly, MitoQ also suppressed mtROS generation in a dose-dependent manner (Figure 6D). These results further confirmed the potential of MitoQ for the treatment of acute colonic injury by reducing oxidative stress and inflammatory cytokines.

## Discussion

Here, we for the first time show that administration of MitoQ decreases the severity of DSS-induced colitis in mice. In addition, scavenging mtROS by MitoQ resulted in a significant decrease of IL-1 beta and IL-18 in DSS-induced colitis. Consequently, our results provide insights into the role of mtROS in the pathogenesis of IBD, and suggest that MitoQ may have therapeutic potential as a new treatment for human IBD.

Oxidative injury induced by increased ROS levels has been demonstrated in patients with IBD and in experimental animals [21]. Also, a randomized controlled trial reported that supplementation of antioxidants resulted in significant clinical improvement of patients with UC [38], indicating that ROS may have a causative role in IBD. By contrast, other randomized controlled trials showed that antioxidant supplementation has no effect on activity of the disease. The reasons for this discrepancy in patients with IBD remain unknown. Since the treatment outcomes of antioxidants are affected by several factors including dosage, duration, phase of the disease, and antioxidant potency [39,40], it may not be easy to clarify reasons for the contrasting results in clinical trials. A number of different cellular sources of ROS such as NADPH oxidase, inducible nitric oxide synthase, myeloperoxidase and xanthine oxidase have already been reported [41,42]. Here, we propose that mitochondria are the major source of ROS generation in IBD, and thus targeting mtROS may be important for understanding the therapeutic efficacy of antioxidants in patients with IBD. The reasons are as follows: first, we revealed that mtROS generation are significantly increased and expression levels of mtETC components are altered in the MNC of patients with IBD, all of which can be normalized after treatment with standard medications (Figure 1); second, we revealed that ROS levels and oxidative injury are increased in DSS-induced mouse colitis, but suppressed by treatment with MitoQ (Figures 3B,C and 4D); finally, other reports also suggested that mitochondrial alterations are important for IBD and CD, including the morphological changes of mitochondria in enterocytes of patients with IBD [43], the inhibited mitochondrial membrane potential in immune peripheral cells of patients with CD, and the functional defects at complex III and IV in isolated muscle mitochondria [23]. In addition, there are many evidences for a role of mtROS in hypoxia during inflammation [44]. Mucosal inflammation including vasculitis, vasoconstriction, thrombosis and edema contributes to inflammatory hypoxia in the intestine. Adaptation to hypoxia relies on the hypoxia-inducible factor, which in turn contributes to the induction of mucosal barrier genes [45,46]. But hypoxia also leads to increased expression of pro-inflammatory mediators [47,48] and increased generation of mtROS [49], and could promote tumor growth [50,51]. Although physiological level of ROS is important for hypoxia-inducible factor stabilization and phagocytosis, ROS are also considered to be second messengers for mucosal injury during IBD. In addition, inhibition of mtROS resulted in restoration of regulatory T cells induction [52]. These findings suggest that prolonged hypoxia can drive a robust inflammatory response that contributes to hypoxia-induced inflammation. Therefore, suppressing overgenerated mtROS might help to attenuate intestinal inflammation by reducing hypoxia and controlling T cell activation.

MitoQ is a well-established mitochondria-targeted antioxidant, and consists of a lipophilic TPP cation covalently linked to ubiquinone, which is the active antioxidant moiety of coenzyme Q. The adsorbed MitoQ in the mitochondrial inner membrane acts as an antioxidant and ubiquinone is rapidly reduced to its active ubiquinol form by complex II. MitoQ shows good pharmacokinetic behavior and was safely administered as a daily oral tablet to patients for a year in phase 2 trials [53]. Furthermore, it has been shown to have good antioxidative, anti-inflammatory and anti-apoptotic effects in many *in vivo* and *in vitro* studies [24-29]. In this study, we showed that MitoQ significantly improves clinical and histological changes in the DSS-induced mouse model of colitis (Figure 2A-G) by reducing oxidative stress and restoring mitochondrial alterations (Figure 3). These results suggest that mtROS may play an important role in IBD and indicate that MitoQ is a promising candidate for treatment of human IBD.

IL-1 beta and IL-18 are members of the IL-1 family of cytokines, which play major roles in the pathogenesis of IBD. Inflammatory cytokine IL-18 induces IL-1 beta, TNF-α and IFN-γ, and thus leads to severe gut inflammation [54]. IL-1 beta increases intestinal permeability [55] and promotes Th17 responses in the gut [10]. Such roles for IL-1 beta and IL-18 in IBD are supported by several studies. For example, it was reported that blockage of IL-1 beta [56,57] or neutralization of IL-18 [58,59] reduces intestinal inflammation. Additionally, homozygous knock-out of NLRP3 and caspase-1 genes, or inhibition of caspase-1 by a specific inhibitor, protects mice from DSS-induced colitis [16,17,60,61]. The precursors of IL-1 beta and IL-18 are cleaved and activated by the cytosolic caspase-1 activating NLRP3 inflammasome, whose physiological activation may be critical in the maintenance of intestinal homeostasis. However, excessive activation of NLRP3 inflammasome leads to severe pathology. NLRP3 inflammasome activation is mediated by ROS via the ROS-sensitive ligand, TXNIP [20]. ROS induce the dissociation of TXNIP from TRX and allow TXNIP to bind NLRP3. Although there is still controversy about the source of ROS responsible for NLRP3 inflammasome activation, our results suggest that mtROS are responsible for its activation. Consistent with this, recent studies revealed that inflammasome activation was observed in mice lacking NADPH oxidase subunits, and in patients with chronic granulomatous disease characterized by defects in NADH-oxidase subunits [62]. In the present study, we showed that MitoQ suppresses ROS-promoted dissociation of TXNIP from TRX, inhibits the interaction between TXNIP and NLRP3 (Figure 4), and significantly decreases levels of IL-1 beta and IL-18 in the colons of mice with DSS-induced colitis (Figure 5A,B). Furthermore, *in vitro* studies demonstrated that MitoQ also suppresses the release of IL-1 beta and IL-18 from human THP-1 cells (Figure 6A,B).

**Figure 7:**
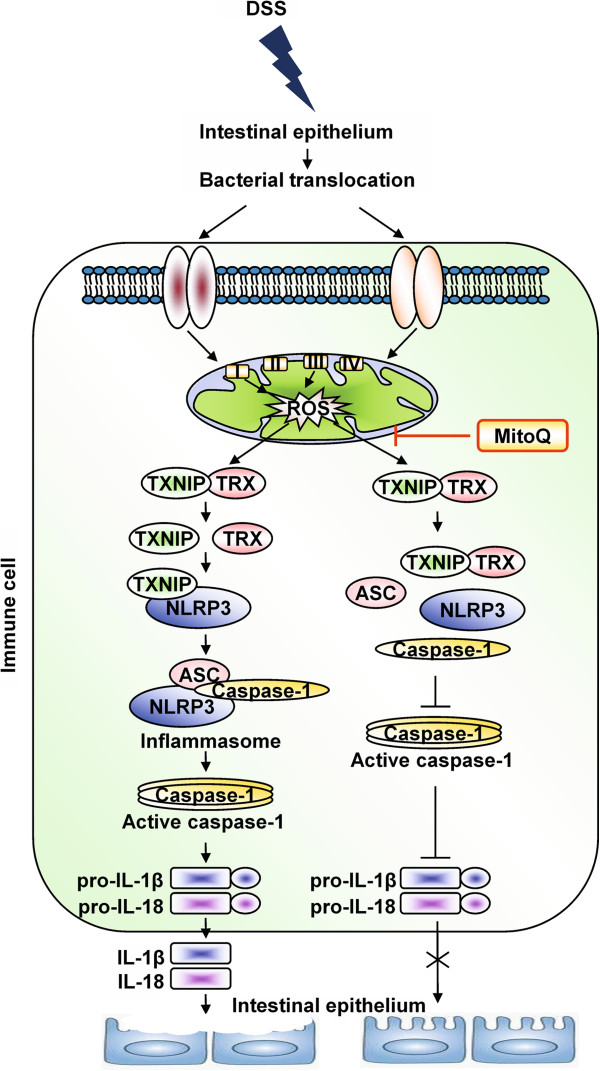
**Schematic representation of the mechanism of action of MitoQ during colitis.** Increased generation of mtROS in the damaged epithelium and activated macrophages leads to dissociation of TXNIP from the TXNIP-TRX complex. Dissociated TXNIP binds to NLRP3 protein and activates the NLRP3 inflammasome complex, which is responsible for cleavage of pro-inflammatory cytokines pro-IL-1 beta and pro-IL-18 into their active forms, thereby increasing intestinal permeability and tissue injury. Moreover, the NLRP3 inflammasome complex activates the adaptive immune system and exacerbates inflammation. Suppressing mtROS with MitoQ can suppress this pathway and inhibit cytokines release, thereby ameliorating inflammation during colitis.

Finally, we conclude that overgeneration of mtROS during IBD leads to increase of inflammatory cytokines IL-1 beta and IL-18 via activation of the NLRP3 inflammasome. Active inflammatory cytokines increase intestinal permeability, tissue injury and decreasing mtROS with MitoQ can suppress this pathway and ameliorate inflammation during colitis (Figure 7).

We here used DSS-induced acute colitis, because it is one of the easiest, time- and cost-saving animal models. Actually, DSS has a direct toxic effect on colonic epithelium leading to a leaky tight junction and bacterial translocation. Therefore, this animal model may reflect an acute injury model rather than an inflammatory disease, indicating that it has a limitation to be used as a good IBD model [63]. Currently, more than 60 animal models are available for the study of IBD, but no individual model can fully reflect human IBD [64,65]. In some cases, investigators have used IL-10 knock-out or TNF (ARE)-deleted mice to study the mechanisms of IBD pathogenesis [66-69]. We have first shown the therapeutic effect of MitoQ on IBD using the DSS-induced acute colitis model. To clarify MitoQ effect on IBD more clearly, additional tests will be needed in other well-established animal models such as IL-10 knock-out mice. If carefully designed studies show the positive effect of MitoQ on IBD treatment in diverse animal models, MitoQ may ultimately be tested in human patients with IBD.

## Conclusion

We suggest that mtROS are an important causative factor in the pathogenesis of IBD. We showed that MitoQ ameliorates acute colonic injury in a mouse model of colitis not only by its antioxidative effects but also by anti-inflammatory effects that suppress the maturation of pro-inflammatory cytokines IL-1 beta and IL-18. Considering the potent protective role of MitoQ in an experimental model of colitis and its proven safety in human clinical trials, MitoQ is a possible therapeutic molecule for the treatment of acute phases of IBD.

## Abbreviations

ASC: Apoptosis-associated speck-like protein containing a CARD; CD: Crohn’s disease; DCF-DA: Dichlorofluorescein-diacetate; DSS: Dextran sulphate sodium; dTPP: Decyltriphenylphosphonium bromide; ELISA: Enzyme-linked immunosorbent assay; Foxp3: Forkhead box p3; IBD: Inflammatory bowel disease; IFNγ: Interferon gamma; IL: Interleukin; IP: Immunoprecipitation; MNC: Mononuclear cell; mtETC: Mitochondrial electron transport chain; mtROS: Mitochondrial reactive oxygen species; PBS: Phosphate-buffered saline; ROS: Reactive oxygen species; RT-PCR: Reverse transcription polymerase chain reaction; SE: Standard error; TNFα: Tumor necrosis factor alpha; TPP: Triphenylphosphonium; TRX: Thioredoxin; TXNIP: Thioredoxin interacting protein; UC: Ulcerative colitis; WT: Wild type.

## Competing interests

MPM is on the scientific advisory board of Antipodean Pharmaceuticals, which is developing MitoQ as a therapy. All other authors declare that they have no competing interests.

## Authors’ contributions

AD, SSK: study concept and design; AD, SL, HJK: acquisition of data; AD, SL, SSK: analysis and interpretation of data; AD, SSK: drafting of the manuscript; AD, JKR, SL, AJ, MNN, JH, KSY, HJK, JHP, MPM, SSK: critical revision of the manuscript for intellectual content; MPM: material support; SSK: study supervision. All authors read and approved the final manuscript.

## Pre-publication history

The pre-publication history for this paper can be accessed here:

http://www.biomedcentral.com/1741-7015/11/178/prepub

## Supplementary Material

Additional file 1: Table S1 Medications for patients with IBD. Click here for file

## References

[B1] Kethu SR Extraintestinal manifestations of inflammatory bowel diseases J Clin Gastroenterology 2006 40 467 475 10.1097/00004836-200607000-00003 16825927

[B2] Guidi L Pugliese D Armuzzi A Update on the management of inflammatory bowel disease: specific role of adalimumab Clin Exp Gastroenterol 2011 4 163 172 21904462 10.2147/CEG.S14558 PMC3163921

[B3] Naija N Karoui S Serghini M Kallel L Boubaker J Filali A Management of failure of infliximab in inflammatory bowel disease Tunis Med 2011 89 517 521 21681712

[B4] Molodecky NA Soon IS Rabi DM Ghali WA Ferris M Chernoff G Benchimol EI Panaccione R Ghosh S Barkema HW Kaplan GG Increasing incidence and prevalence of the inflammatory bowel diseases with time, based on systematic review Gastroenterology 2012 142 46 54 10.1053/j.gastro.2011.10.001 22001864

[B5] Maloy KJ Powrie F Intestinal homeostasis and its breakdown in inflammatory bowel disease Nature 2011 474 298 306 10.1038/nature10208 21677746

[B6] Dinarello CA Immunological and inflammatory functions of the interleukin-1 family Annu Rev Immunol 2009 27 519 550 10.1146/annurev.immunol.021908.132612 19302047

[B7] Chung Y Chang SH Martinez GJ Yang XO Nurieva R Kang HS Ma L Watowich SS Jetten AM Tian Q Dong C Critical regulation of early Th17 cell differentiation by interleukin-1 signaling Immunity 2009 30 576 587 10.1016/j.immuni.2009.02.007 19362022 PMC2705871

[B8] Clambey ET McNamee EN Westrich JA Glover LE Campbell EL Jedlicka P de Zoeten EF Cambier JC Stenmark KR Colgan SP Eltzschig HK Hypoxia-inducible factor-1 alpha-dependent induction of FoxP3 drives regulatory T-cell abundance and function during inflammatory hypoxia of the mucosa Proc Natl Acad Sci U S A 2012 109 E2784 E2793 10.1073/pnas.1202366109 22988108 PMC3478644

[B9] Ziegler SF Buckner JH FOXP3 and the regulation of Treg/Th17 differentiation Microbes Infect 2009 11 594 598 10.1016/j.micinf.2009.04.002 19371792 PMC2728495

[B10] Coccia M Harrison OJ Schiering C Asquith MJ Becher B Powrie F Maloy KJ IL-1 beta mediates chronic intestinal inflammation by promoting the accumulation of IL-17A secreting innate lymphoid cells and CD4 (+) Th17 cells J Exp Med 2012 209 1595 1609 10.1084/jem.20111453 22891275 PMC3428945

[B11] Mahida YR Wu K Jewell DP Enhanced production of interleukin 1-beta by mononuclear cells isolated from mucosa with active ulcerative colitis of Crohn's disease Gut 1989 30 835 838 10.1136/gut.30.6.835 2787769 PMC1434123

[B12] McAlindon ME Hawkey CJ Mahida YR Expression of interleukin 1 beta and interleukin 1 beta converting enzyme by intestinal macrophages in health and inflammatory bowel disease Gut 1998 42 214 219 10.1136/gut.42.2.214 9536946 PMC1726995

[B13] Guimbaud R Bertrand V Chauvelot-Moachon L Quartier G Vidon N Giroud JP Couturier D Chaussade S Network of inflammatory cytokines and correlation with disease activity in ulcerative colitis Am J Gastroenterol 1998 93 2397 2404 10.1111/j.1572-0241.1998.00694.x 9860399

[B14] Schoultz I Verma D Halfvarsson J Törkvist L Fredrikson M Sjöqvist U Lördal M Tysk C Lerm M Söderkvist P Söderholm JD Combined polymorphisms in genes encoding the inflammasome components NALP3 and CARD8 confer susceptibility to Crohn’s disease in Swedish men Am J Gastroenterol 2009 104 1180 1188 10.1038/ajg.2009.29 19319132

[B15] Schroder K Tschopp J The inflammasome. Cell 2010 140 821 832 10.1016/j.cell.2010.01.040 20303873

[B16] Bauer C Duewell P Mayer C Lehr HA Fitzgerald KA Dauer M Tschopp J Endres S Latz E Schnurr M Colitis induced in mice with dextran sulfate sodium (DSS) is mediated by the NLRP3 inflammasome Gut 2010 59 1192 1199 10.1136/gut.2009.197822 20442201

[B17] Bauer C Duewell P Lehr HA Endres S Schnurr M Protective and aggravating effects of Nlrp3 inflammasome activation in IBD models: influence of genetic and environmental factors Dig Dis 2012 30 1 82 90 23075874 10.1159/000341681

[B18] Shimada K Crother TR Karlin J Dagvadorj J Chiba N Chen S Ramanujan VK Wolf AJ Vergnes L Ojcius DM Rentsendorj A Vargas M Guerrero C Wang Y Fitzgerald KA Underhill DM Town T Arditi M Oxidized mitochondrial DNA activates the NLRP3 inflammasome during apoptosis Immunity 2012 36 401 414 10.1016/j.immuni.2012.01.009 22342844 PMC3312986

[B19] Zhou R Yazdi AS Menu P Tschopp J Role for mitochondria in NLRP3 inflammasome activation Nature 2011 469 221 225 10.1038/nature09663 21124315

[B20] Zhou R Tardivel A Thorens B Choi I Tschopp J Thioredoxin-interacting protein links oxidative stress to inflammasome activation Nature Immunol 2010 11 136 140 10.1038/ni.1831 20023662

[B21] Pavlick KP Laroux FS Fuseler J Wolf RE Gray L Hoffman J Grisham MB Role of reactive metabolites of oxygen and nitrogen in inflammatory bowel disease Free Radic Biol Med 2002 33 311 322 12126753 10.1016/s0891-5849(02)00853-5

[B22] Beltrán B Nos P Dasí F Iborra M Bastida G Martínez M O'Connor JE Sáez G Moret I Ponce J Mitochondrial dysfunction, persistent oxidative damage, and catalase inhibition in immune cells of naive and treated Crohn's disease Inflamm Bowel Dis 2010 16 76 86 10.1002/ibd.21027 19637347

[B23] Restivo NL Srivastava MD Schafer IA Hoppel CL Mitochondrial dysfunction in a patient with Crohn’s disease: possible role in pathogenesis J Pediatr Gastroenterol Nutr 2004 38 534 538 10.1097/00005176-200405000-00014 15097444

[B24] Mukhopadhyay P Horváth B Zsengellėr Z Bátkai S Cao Z Kechrid M Holovac E Erdėlyi K Tanchian G Liaudet L Stillman IE Joseph J Kalyanaraman B Pacher P Mitochondrial reactive oxygen species generation triggers inflammatory response and tissue injury associated with hepatic ischemia-reperfusion: therapeutic potential of mitochondrially targeted antioxidants Free Radic Biol Med 2012 53 1123 1138 10.1016/j.freeradbiomed.2012.05.036 22683818 PMC3432152

[B25] Graham D Huynh NN Hamilton CA Beattie E Smith RA Cochemé HM Murphy MP Dominiczak AF Mitochondria-targeted antioxidant MitoQ10 improves endothelial function and attenuates cardiac hypertrophy Hypertension 2009 54 322 328 10.1161/HYPERTENSIONAHA.109.130351 19581509

[B26] Supinski GS Murphy MP Callahan LA MitoQ administration prevents endotoxin-induced cardiac dysfunction Am J Physiol Regul Integr Comp Physiol 2009 297 R1095 R1102 10.1152/ajpregu.90902.2008 19657095 PMC2763820

[B27] Chacko BK Srivastava A Johnson MS Benavides GA Chang MJ Ye Y Jhala N Murphy MP Kalyanaraman B Darley-Usmar VM The mitochondria-targeted ubiquinone MitoQ decreases ethanol-dependent micro and macro hepatosteasis Hepatology 2011 54 153 163 21520201 10.1002/hep.24377 PMC3125473

[B28] Lowes DA Thottakam BM Webster NR Murphy MP Galley HF The mitochondria-targeted antioxidant MitoQ protects against organ damage in a lipopolysaccharide-peptidoglycan model of sepsis Free Radic Biol Med 2008 45 1559 1565 10.1016/j.freeradbiomed.2008.09.003 18845241

[B29] Gane EJ Weilert F Orr DW Keogh GF Gibson M Lockhart MM Frampton CM Taylor KM Smith RA Murphy MP The mitochondria-targeted anti-oxidant mitoquinone decreases liver damage in a phase II study of hepatitis C patients Liver Int 2010 30 1019 1026 10.1111/j.1478-3231.2010.02250.x 20492507

[B30] Lim S Won H Kim Y Jang M Jyothi KR Kim Y Dandona P Ha J Kim SS Antioxidant enzymes induced by repeated intake of excess energy in the form of high-fat, high-carbohydrate meals are not sufficient to block oxidative stress in healthy lean individuals Br J Nutr 2011 106 1544 1551 10.1017/S0007114511002091 21676280

[B31] Lee S Tak E Lee J Rashid MA Murphy MP Ha J Kim SS Mitochondrial H2O2 generated from electron transport chain complex I stimulates muscle differentiation Cell Res 2011 21 817 834 10.1038/cr.2011.55 21445095 PMC3203677

[B32] Yan Y Kolachala V Dalmasso G Nguyen H Laroui H Sitaraman SV Merlin D Temporal and spatial analysis of clinical and molecular parameters in dextran sodium sulfate induced colitis Plos One 2009 4 6 e6073 e6080 10.1371/journal.pone.0006073 19562033 PMC2698136

[B33] Yotsuya S Shikama H Imamura M Efficacy of the inflammatory cell infiltration inhibitor IS-741 on colitis induced by dextran sulfate sodium in the rat Jpn J Pharmacol 2001 87 151 157 10.1254/jjp.87.151 11700014

[B34] Adlam VJ Harrison JC Porteous CM James AM Smith RA Murphy MP Sammut IA Targeting an antioxidant to mitochondria decreases cardiac ischemia-reperfusion injury FASEB J 2005 19 1088 1095 10.1096/fj.05-3718com 15985532

[B35] Hayakawa Y Hirata Y Nakagawa H Sakamoto K Hikiba Y Otsuka M Ijichi H Ikenoue T Tateishi K Akanuma M Ogura K Yoshida H Ichijo H Omata M Maeda S Apoptosis signal-regulating kinase 1 regulates colitis and colitis-associated tumorigenesis by the innate immune responses Gastroenterology 2010 138 1055 1067 10.1053/j.gastro.2009.11.015 19931259

[B36] Nishiyama A Matsui M Iwata S Hirota K Masutani H Nakamura H Takagi Y Sono H Gon Y Yodoi J Identification of thioredoxin-binding protein -2/vitamin D3 upregulated protein 1 as a negative regulator of thioredoxin function and expression J Biol Chem 1999 274 21645 21650 10.1074/jbc.274.31.21645 10419473

[B37] van de Veerdonk FL Netea MG Dinarello CA Joosten LA Inflammasome activation and IL-1 beta and IL-18 processing during infection Trends Immunol 2011 32 110 116 10.1016/j.it.2011.01.003 21333600

[B38] Seidner DL Lashner BA Brzezinski A Banks PL Goldblum J Fiocchi C Katz J Lichtenstein GR Anton PA Kam LY Garleb KA Demichele SJ An oral supplement enriched with fish oil, soluble fiber, and antioxidants for corticosteroid sparing in ulcerative colitis: a randomized, controlled trial Clin Gastroenterol Hepatol 2005 3 358 369 10.1016/S1542-3565(04)00672-X 15822041

[B39] Aghdassi E Wendland BE Steinhart AH Wolman SL Jeejeebhoy K Allard JP Antioxidant vitamin supplementation in Crohn’s disease decreases oxidative stress: a randomized controlled trial Am J Gastroenterol 2003 98 348 353 12591053 10.1111/j.1572-0241.2003.07226.x

[B40] Geerling BJ Badart-Smook A van Deursen C van Houwelingen AC Russel MG Stockbrügger RW Brummer RJ Nutritional supplementation with N-3 fatty acids and antioxidants in patients with Crohn’s disease in remission: effects on antioxidant status and fatty acid profile Inflamm Bowel Dis 2000 6 77 84 10.1097/00054725-200005000-00002 10833065

[B41] Hausmann M Spöttl T Andus T Rothe G Falk W Schölmerich J Herfarth H Rogler G Subtractive screening reveals up-regulation of NADPH oxidase expression in Crohn's disease intestinal macrophages Clin Exp Immunol 2001 125 48 55 10.1046/j.1365-2249.2001.01567.x 11472425 PMC1906098

[B42] Szanto I Rubbia-Brandt L Kiss P Steger K Banfi B Kovari E Herrmann F Hadengue A Krause KH Expression of NOX1, a superoxide-generating NADPH oxidase, in colon cancer and inflammatory bowel disease J Pathol 2005 207 164 176 10.1002/path.1824 16086438

[B43] Söderholm JD Olaison G Peterson KH Franzén LE Lindmark T Wirén M Tagesson C Sjödahl R Augmented increase in tight junction permeability by luminal stimuli in the non-inflamed ileum of Crohn’s Disease Gut 2002 50 307 313 10.1136/gut.50.3.307 11839706 PMC1773145

[B44] Cash TP Pan Y Simon MC Reactive oxygen species and cellular oxygen sensing Free Radic Biol Med 2007 43 1219 1225 10.1016/j.freeradbiomed.2007.07.001 17893032 PMC2696222

[B45] Eltzschig HK Carmeliet P Hypoxia and inflammation N Engl J Med 2011 364 656 665 10.1056/NEJMra0910283 21323543 PMC3930928

[B46] Eltzschig HK Sitkovsky MV Robson SC Purinergic signaling during inflammation N Engl J Med 2013 368 1260 23534573 10.1056/NEJMc1300259

[B47] Shah YM Ito S Morimura K Chen C Yim SH Haase VH Gonzalez FJ Hypoxia-inducible factor augments experimental colitis through a MIF-dependent inflammatory signaling cascade Gastroenterology 2008 134 2036 2048 10.1053/j.gastro.2008.03.009 18439915 PMC2533811

[B48] Scortegagna M Cataisson C Martin RJ Hicklin DJ Schreiber RD Yuspa SH Arbeit JM HIF-1alpha regulates epithelial inflammation by cell autonomous NFkB activation and paracrine stromal remodeling Blood 2008 111 3343 3354 10.1182/blood-2007-10-115758 18199827 PMC2275004

[B49] Lluis JM Buricchi F Chiarugi P Morales A Fernandez-Checa JC Dual role of mitochondrial reactive oxygen species in hypoxia signaling: activation of nuclear factor-{kappa}B via c-SRC and oxidant-dependent cell death Cancer Res 2007 67 7368 7377 10.1158/0008-5472.CAN-07-0515 17671207

[B50] Semenza GL Defining the role of HIF-1 in cancer biology and therapeutics Oncogene 2010 29 625 634 10.1038/onc.2009.441 19946328 PMC2969168

[B51] Xue X Taylor M Anderson E Hao C Qu A Greenson JK Zimmermann EM Gonzalez FJ Shah YM Hypoxia-inducible factor 2 alpha activation promotes colorectal cancer progression by dysregulation iron homeostasis Cancer Res 2012 72 2285 2293 10.1158/0008-5472.CAN-11-3836 22419665 PMC3342485

[B52] Kaminski MM Sauer SW Klemke CD Süss D Okun JG Krammer PH Gülow K Mitochondrial reactive oxygen species control T cell activation by regulating IL-2 and IL-4 expression: mechanism of ciprofloxacin-mediated immunosuppression J Immunol 2010 184 4827 4841 10.4049/jimmunol.0901662 20335530

[B53] Snow BJ Rolfe FL Lockhart MM Frampton CM O'Sullivan JD Fung V Smith RA Murphy MP Taylor KM Protect Study Group A double-blind, placebo-controlled study to assess the mitochondria-targeted antioxidant MitoQ as a disease modifying therapy in Parkinson’s disease Mov Disord 2010 25 1670 1674 10.1002/mds.23148 20568096

[B54] Pagès F Lazar V Berger A Danel C Lebel-Binay S Zinzindohoué F Desreumaux P Cellier C Thiounn N Bellet D Cugnenc PH Fridman WH Analysis of interleukin-18, interleukin-1 converting enzyme (ICE) and interleukin-18-related cytokines in Crohn's disease lesions Eur Cytokine Netw 2001 12 97 104 11282552

[B55] Al-Sadi R Guo S Dokladny K Smith MA Ye D Kaza A Watterson DM Ma TY Mechanism of interleukin-1beta induced-increase in mouse intestinal permeability in vivo J Interferon Cytokine Res 2012 32 474 484 10.1089/jir.2012.0031 22817402 PMC3464071

[B56] Thomas TK Will PC Srivastava A Wilson CL Harbison M Little J Chesonis RS Pignatello M Schmolze D Symington J Kilian PL Thompson RC Evaluation of an interleukin-1 receptor antagonist in the rat acetic acid-induced colitis model Agents Actions 1991 34 187 190 10.1007/BF01993274 1838896

[B57] Cominelli F Nast CC Duchini A Lee M Recombinant interleukin-1 receptor antagonist blocks the pro-inflammatory activity of endogenous interleukin-1 in rabbit immune colitis Gastroenterology 1992 103 65 71 1535326 10.1016/0016-5085(92)91096-m

[B58] Sivakumar PV Westrich GM Kanaly S Garka K Born TL Derry JM Viney JL Interleukin 18 is a primary mediator of the inflammation associated with dextran sulphate sodium induced colitis: blocking interleukin 18 attenuates intestinal damage Gut 2002 50 812 820 10.1136/gut.50.6.812 12010883 PMC1773244

[B59] Ten Hove T Corbaz A Amitai H Aloni S Belzer I Graber P Drillenburg P van Deventer SJ Chvatchko Y Te Velde AA Blockade of endogenous IL-18 ameliorates TNBS-induced colitis by decreasing local TNF-alpha production in mice Gastroenterology 2001 121 1372 1379 10.1053/gast.2001.29579 11729116

[B60] Siegmund B Lehr HA Fantuzzi G Dinarello CA IL-1 beta-converting enzyme (caspase-1) in intestinal inflammation Proc Natl Acad Sci U S A 2001 98 13249 13254 10.1073/pnas.231473998 11606779 PMC60856

[B61] Loher F Bauer C Landauer N Schmall K Siegmund B Lehr HA Dauer M Schoenharting M Endres S Eigler A The interleukin-1 beta-converting enzyme inhibitor pralnacasan reduces dextran sulfate sodium-induced murine colitis and T helper 1 T-cell activation J Pharmacol Exp Ther 2004 308 583 590 14610233 10.1124/jpet.103.057059

[B62] Meissner F Seger RA Moshous D Fischer A Reichenbach J Zychlinsky A Inflammasome activation in NADPH oxidase defective mononuclear phagocytes from patients with chronic granulomatous disease Blood 2010 116 1570 1573 10.1182/blood-2010-01-264218 20495074 PMC2938844

[B63] Ni J Chen SF Hollander D Effects of dextran sulphate sodium on intestinal epithelial cells and intestinal lymphocytes Gut 1996 39 234 241 10.1136/gut.39.2.234 8991862 PMC1383305

[B64] Mizoguchi A Animal models of inflammatory bowel disease Prog Mol Biol Transl Sci 2012 105 263 320 22137435 10.1016/B978-0-12-394596-9.00009-3

[B65] Mizoguchi A Mizoguchi E Inflammatory bowel disease, past, present and future: lessons from animal models J Gastroenterol 2008 43 1 17 10.1007/s00535-007-2111-3 18297430

[B66] Berg DJ Zhang J Weinstock JV Ismail HF Earle KA Alila H Pamukcu R Moore S Lynch RG Rapid development of colitis in NSAID-treated IL-10-deficient mice Gastroenterology 2002 123 1527 1542 10.1053/gast.2002.1231527 12404228

[B67] Kühl AA Pawlowski NN Grollich K Loddenkemper C Zeitz M Hoffmann JC Aggravation of intestinal inflammation by depletion/deficiency of gammadelta T cells in different types of IBD animal models J Leukoc Biol 2007 81 168 175 17041003 10.1189/jlb.1105696

[B68] Huybers S Apostolaki M van der Eerden BC Kollias G Naber TH Bindels RJ Hoenderop JG Murine TNF(DeltaARE) Crohn's disease model displays diminished expression of intestinal Ca2+ transporters Inflamm Bowel Dis 2008 14 803 811 10.1002/ibd.20385 18266230

[B69] Hale LP Greer PK A novel murine model of inflammatory bowel disease and inflammation-associated colon cancer with ulcerative colitis-like features PLoS One 2012 7 e41797 e41808 10.1371/journal.pone.0041797 22848611 PMC3407062

