# Supplementary material for: Mitochondria-targeted antioxidant MitoQ ameliorates experimental mouse colitis by suppressing NLRP3 inflammasome-mediated inflammatory cytokines
Source: BMC Med. 2013 Aug 6;11:178. doi: 10.1186/1741-7015-11-178 (PMC3750576; doi:10.1186/1741-7015-11-178)
Supplement: Additional file 1: Table S1 — Medications for patients with IBD. [file 1741-7015-11-178-S1.doc]

**Supplementary table 1. Medications for patients with IBD**

| **Patients**  **number** | **Sex** | **Age** | **Diagnosis** | **Medications** | | | | |
| --- | --- | --- | --- | --- | --- | --- | --- | --- |
|  |  |  |  | **5-ASA** | **Steroid** | **Azathioprine** | **Mercapto-purine** | **Remicade** |
| **1** | M | 18 | CD | + | - | + | - | + |
| **2** | M | 24 | UC | + | + | + | - | + |
| **3** | M | 30 | UC | + | + | + | + | - |
| **4** | F | 32 | UC | + | + | + | - | - |
| **5** | M | 28 | UC | + | + | + | - | - |
| **6** | M | 31 | CD | + | + | + | - | - |
| **7** | M | 26 | UC | + | + | - | - | - |
| **8** | F | 29 | UC | + | + | + | - | + |
| **9** | M | 23 | CD | + | + | + | - | - |
| **10** | M | 32 | UC | + | + | + | - | - |
| **11** | M | 18 | CD | + | + | + | - | + |
| **12** | M | 43 | CD | + | + | + | - | - |
| **13** | F | 36 | UC | + | + | + | - | - |
| **14** | F | 32 | UC | + | + | + | - | - |

**UC –** Ulcerative colitis

**CD –** Crohn’s disease
